# The Organs of Sensation and Motion Discovered

**Published:** 1864-01

**Authors:** W. Byrd Powell

**Affiliations:** Covington, Ky.


### CHICAGO MEDICAL EXAMINER.

N. S. DAVIS, M.D., Editor.

VOL. V. JANUARY, 1864. NO. 1.

### Original Contributions.

### ARTICLE I.

### THE ORGANS OF SENSATION AND MOTION DISCOVERED.

By W. BYRD POWELL, M.D., of Covington, Ky.

Mr Editor:—Believing that the discovery of the organs of sensation and motion, or the sensory and motory centres, will be interesting, if not useful, to the profession, I send for the Examiner a contribution on these subjects. Physiologists have long, if not always, spoken of the sensory and motory centres; but hitherto they have known nothing of their respective organs. Physiologists have conclusively arrived at the conviction that the faculties of sensation and motion are independent of each other. But are they primitive and independent faculties? I do not remember to have found this question answered—it has only, I believe, been assumed. I will now show that the affirmative is true. Dr. Spurzhiem, high authority, presents it as a rule, that when a faculty is more strongly manifested by one sex than the other, or by one people or nation than another, it is primitive and independent. Men are more sensual, generally, than women. Men complain more than women under an exposure to cold, and under the inflictions of the surgeon's knife, than women do. The white variety, at least, of the Caucasian species of our genus is greatly more capable of suffering than is the American species; our Indians bear torture with comparative impunity. From these facts, I infer that animal sensibility is a primitive and independent faculty.

*Muscular motion.* As the males of all known species of animals are more muscular than the females, so I infer that this faculty is both primitive and independent.

Of the functions of the cerebellum nothing, I believe, was definitely known before the investigations of Dr. GALL. He discovered it to be the source of the "instinct of reproduction;" but this opinion has been ably but unsuccessfully controverted. Since the demise of this distinguished observer and physiologist, some French physiologists, for the purpose of disproving Dr. GALL'S conclusion in relation to the function of the cerebellum, obtained the cerebelli of stallions, geldings, and mares and weighed them. They found those of some mares and geldings to weigh more than those of some stallions. Dr. GALL did not teach that the "instinct of reproduction" was the exclusive function of the cerebellum, nor did he teach the contrary. He taught that animals having a large and broad cerebellum were the most successful procreators; but he never comprehended why it was so. He thus left the impression that the entire cerebellum was the organ of the "instinct of reproduction." But this conclusion does not necessarily follow; because other faculties may be essential to reproduction than the instinct of reproduction. The existence of the organs of other faculties in the cerebellum may explain the fact that some mares and geldings may have a larger cerebellum than some stallions; the sequel will show. These physiologists, if I mistake not, entertained the opinion that the coördination and regulation of muscular movements constituted the function of the cerebellum, a most preposterous opinion, certainly; in as much as these functions are purely intellectual. Is there any common sense in the idea that the muscular movements of the pianist, violinist, and engraver are the results of a blind instinct? Some other French physiologists taught that the cerebellum was the organ of sensation. The sequel will show that the cerebellum comprises the organs of sensation, motion, and the "instinct of reproduction."

As my observations in relation to sensation and motion were conducted *pari passu*, I find it more convenient to treat of them together. As the history of these discoveries is interesting, and shows how they were suggested, I will introduce it.

In 1837, during the vacation of the Medical College of Louisiana, New Orleans, I visited, as a phrenological teacher, the City of Claiborne, Ala., and in an hour or so after my arrival, Dr. JOHNSON, with four other gentlemen, called on me. The Dr. had with him a human skull, and as he approached me, he said, "Prof. Powell, I will bet you ten dollars you cannot indicate the character of the man representod by this skull, by an examination of it." As he was to me a stranger, and not being pleased with his introduction, I resolved to parry him. I rejoined, as I know I can, and you do not know that I can, the chances are all in my favor; and, therefore, Sir, I cannot, as an honest man, accept your proposition. He rejoined, "But I am willing to risk it." I rejoined, I think it very probable that you know but little of the science of phrenology, and still less of my ability in the application of it; therefore, your willingness to risk, under such circumstances, affords me no relief; consequently, I must decline the acceptance of your proposition.

At this moment, one of the other gentlemen said, "Professor, you will greatly oblige us by indicating the character of the man of whom that is the skull?" I responded, I will, Sir, if Dr. JOHNSON will make a memorandum of the leading peculiarities of his character, and the principal events of his history. The Dr. rejoined, "I will do it, Sir." He did it; and at my request he gave it to the gentleman who made the request that I should give my opinion of the man, to keep till I should have expressed my opinion. I took the skull and remarked thus:—The man represented by this skull was tall and slender; his hair and eyes were brown; his complexion dark, or in other words, his temperament was bilious encephalic. (The Dr. ejaculated, "I did not mention that in the memorandum; but, Sir, you are graphically correct.") In relation to his character, I have to say, that a more degraded specimen of our species can scarcely be found in any civilized community than he must have been, for he observed none of the proprieties incidental to civil society. He was very cowardly; and equally negligent of all the duties incidental to humanity, in the social state; and yet he was respectably endowed with mechanical and mathematical abilities; but he was so irresponsible, and destitute of industrial energy, that they could have been of but little use to himself or any one else; but the governing proclivity of his nature was his sexual propensity, and it was so exceedingly importunate that I cannot believe that he was capable of restraining it within legal limits. I now said, I have concluded, gentlemen. The Dr. rejoined, "If you had accepted my proposition, I would now be minus ten dollars."

The memorandum was now handed to me, and it read thus: —"The man represented by the skull in question was known as Thomas Kennedy, a native of Annapolis, Md.; he was by trade a house carpenter, and esteemed a good one; he was also a practical surveyor; but he was so unreliable and worthless that he was but rarely employed to do anything; he was so cowardly that the boys bullied over him in the streets. At the age of 14 years, he attempted the violation of a girl 7 years old; at the age of 18 years, he attempted a similar outrage upon a girl 9 years old; at the age of 20 years, he married, and a day or so after, his wife arrested him in attempting a similar outrage upon her black maidservant, 8 years old; at the age of 24 years, his wife died, and he went to Virginia, and perpetrated a rape on a girl 10 years old; and for this crime he was hung —while standing on the drop, with the rope about his neck, he informed the spectators that such commerce with female children had been the governing passion of his life."

The Dr. and his friends thought that my delineation of the character of Kennedy was exceedingly accurate, but I was not satisfied; for I did not, and at the time could not, indicate the direction his sexual propensity had taken; and yet I could not doubt that his sexual propensity for female children had a physiological cause, and I desired a knowledge of it, and resolved, if possible, to discover it. His cerebellum was very deep in its mesial position, the condition from which I inferred the importunate character of his amatory propensity,—the organs of which I had previously become fully assured by pathological evidence. Between the mastoid processes his cerebellum was very unusually contracted, and this condition I remembered to have seen in five other men who had perpetrated the same variety of crime, and of two of whom I had the crania, and the other three I saw in state's prisons. Such a uniformity of this condition induced me to suspect that with such men there was an organic defect.

I analyzed the reproductive function, and concluded that sensation and motion were as indispensable as desire; and as he had manifested motor power enough to take his neck to the gallows, and as deficient animal sensibility would, as I conceived, explain his desire for female children, so I thought it probable that his endowment of animal sensibility was greatly insufficient; and this I was inclined to attribute to the small development of the extreme lateral portions of his cerebellum.

Having concluded that the faculties of sensation and motion were primitive and independent; and also arrived at what I deemed a probability as to the nature of his condition; I was prepared to undertake the discovery of the organs of the two faculties above named, but more particularly that of sensation. In addition to the skull of Kennedy, and also those I had of other men, executed for the same variety of crime, the mastoid processes did not stand out boldly, as they do in most human crania; on the contrary, they were depressed, and so was the occipital bone adjoining them, respectively.

My first observation was made on my carriage horses, which were of about the same age, size, color, and keeping. I took them out of a close stable, on a cold and frosty morning, which caused a shivering of the entire surface of one of them, the other appeared as insensible to the atmospheric condition as if he had been dead; this difference arrested my attention, and I immediately examined and compared their heads. The mastoid processes of the sensitive horse stood out boldly from his head, and the adjacent portion of the occipital bone was full; the dedevelopments of the other were precisely the reverse: his mastoids were depressed, and greatly concealed by the soft parts. The development of the two horses were as different as were their manifestations.

I now placed every human head that came into my presence under contribution, when propriety permitted. By this procedure it was but a month or two before I became satisfied that the extreme lateral portions of the cerebellum are the organs of animal sensibility, or, as I understand the subject, they constitute the nerve centre. When these organs are largely developed they cause the mastoid processes to project from the head, and a fulness of the occipital bone interiorly and posteriorly to them, respectively. In living subjects, the cervical muscles which are situated posteriorly to the mastoids, have a full or fleshy appearance when the organs are large. These organs, I infer, are always diseased or subjected to undue pressure in cases of hemiplegia of sensation.

A high development of these organs indicates a tendency of the body to grossness or fulness. Females who adopt the courtesan profession from inclination have a high development of these organs. Their high development is also the cause of nymphomania, I believe. Inveterate masturbators have those organs small, and so have those who have an intemperate desire for commerce with female children. I have the crania of three men, one black and two white, who were executed for rape on little girls, and I have seen several men in state's prisons for the same variety of crime, and all of them had these organs small, and the amatory organs large.

*The faculty of muscular motion.* In my analysis of the reproductive function, I found that, to males at least, muscular motion is as much a *sine qua non* in the reproductive function as the instinct of reproduction or sensation, and hence indispensable to the perpetuity of the species; and hence I inferred it to be probable that its organ would be found in the vicinity of those of sensation and of the reproductive instinct, to coöperate with them. Knowing my carriage horses to be exceeding dissimilar in relation to their muscular qualities—the sensitive one never balked, and the other would do nothing else, except through fear of the whip, consequently, I again examined their heads, and found the former to be very fully developed, about midway between the mesial line and the respective mastoid processes, and the latter to be to an equal degree depressed at the corresponding localities. At this time, I was travelling, and made it a rule to halt every team I met, and learn of the teamster the quality of his respective horses, with reference to draught. By this course, I was, in a few days, able to distinguish, at the distance of several yards, a true from a balky horse,—the faithful horses were uniformly much developed between the mastoid processes and the mesial line. Having thus become familiar with the locality and development of the organs of motion in horses, I directed my attention to men, and in a month, I could distinguish, at sight of the occipital portion of the head and neck, the faithful laborer from the shirker.

These organs are situated midway between the mastoid processes, respectively, and the mesial line, and in proportion to their development, the base of the occipital bone will be downwardly developed, and when large the superior lateral portions of the neck are full and fleshy, and when they are small there is a correspondingly deep depression near the base of the occipital bone, between the mesial and laternal muscles of the neck; and this, I think, to be invariably the case with those who have a scrofulous diathesis. Horses having these organs large are not adapted to the saddle or pleasure carriages—their muscles are heavy and their motions are rough, but for draught they are best. (I will state incidentally, that the mesial third of the cerebellum constitutes the organs of the "instinct of reproduction" or amativeness. The verity of this statement I have had placed beyond any possible doubt by pathological conditions. In the preceding 25 years, I have been consulted by seven men who were, respectively, the subject of severe paroxysms of pain, brought on by excessive commerce with the other sex, with a consequent loss of the amatory propensity. It is proper to add that in these cases, respectively, the organs of sensation and motion were but little developed.) When a man complains of constant soreness and of paroxysmally severe pain on either or both sides of the mesial line, between the occipital protuberance and the foramen magnum, it may be pretty safely inferred that his commerce with the other sex has been intemperate. I have known several stallions and bulls to have lost their ability to procreate, and they were, respectively, very feebly endowed with the organs of sensation and motion. Dr. GALL remarks, "I have always observed that animals having a large and broad cerebellum were the most successful procreators;" but he never comprehended the secret of it, because he knew nothing of the organs of sensation and motion. We can now understand how it may be the fact that a mare or gelding may have a larger cerebellum than a stallion. If the former have large organs of sensation and motion, with even small organs of amativeness, they would have a larger cerebellum than a stallion with large organs of amativeness and small organs of sensation and motion; hence the fact, that some French physiologists found some mares and geldings with a larger cerebellum than had some stallions, did not prove Dr. GALL's conclusion to be an error, that the instinct of reproduction was referable to the cerebellum.

I have labored much to ascertain the essential function of the motory organs, but am not sure that I have succeeded; my conclusion, however, is that they are generators of motor power, for those who are highly endowed with these organs are sustained in their muscular efforts, and readily recover from muscular fatigue.

*The discovery of the organs of sensation and motion demonstrated.* In a little time after becoming confident that I had discovered the organs of sensation and motion, I returned to New Orleans, and in a day or so after my arrival, my colleague, Prof. HARRISON, called on me and solicited my presence at a *post mortem* examination of the brain of a youth of about 17 years of age, who died in the Charity Hospital. When taken to the Hospital, a year before his death, he had hemiplegia of sensation of his right side; he was represented as being an inveterate onanist before his seizure of hemiplegia, of which he did not recover; and three days before his death he was assailed with hemiplegia of motion of his left side. When the Professor had concluded the history of the case, I informed him that I had discovered the organs of sensation and also of motion; he rejoined, "This case may furnish some confirmation of your discoveries."

When we had removed the brain from the skull to a table, I turned it upside down, and indicated to the Professor the organ of sensation of the left side. The first incision by the scalpel disclosed about half a drachm of encysted pus; at the sight of which the Professor exclaimed, "Here is a demonstration of your discovery of the organ of sensation!" I now indicated to him the organ of motion of the right side of the brain, and after a search of a few seconds, he said, "And here is a demonstration of the organ of motion;" it was a tubercle as large as a garden green pea of a bluish-gray color and granulated structure.

It presented no indices of inflammation.

*A pathological fact in relation to animal sensibility.* About a year after making the preceding discoveries, I gave a course of phrenological lectures, in the city of Columbus, Miss., and Dr. M. ESTES was one of my class; a few weeks after the conclusion of my course, and I had left the city, I received from the Doctor the following letter:—

"PROF. POWELL,—I send you a fragment of information, which you will know how to appreciate. One of our citizens left the city, a few days since, on a spree, and after dissipating two or three days, he returned, and sent for me. I found him suffering extreme soreness and pain in a space about as large as a half-dollar, precisely where you locate your newly discovered organ of animal sensibility. The opposite side of his body is so sensitive that he cannot allow his bedclothing to touch it, and the extraction of a hair from the same side of his head gives him very severe pain. Yours, &c. M. ESTES."

*A pathological fact in relation to the organ of motion.* In '55, I was seized with hemiplegia of motion of my left side, and three days subsequently the locality of the organ of motion of the right side of my cerebellum became very sore—not painful; two or three applications of galvanism removed the soreness, but under pressure it continued tender for five years. About a year after all tenderness left the part, even under pressure, a clairvoyant lady called on me, and as a drowning man will grab at a straw, so I requested her to take a peep into my brain, and inform me of its condition? She rose from her chair, walked round to my back, placed her finger on the organ, and said, "In there is a tubercule as large as a grain of black pepper, and of the color of gray limestone, the surface of it appears ragged; I think it is smaller than it has been; and you will again have the use of yourself."

I do not comprehend clairvoyance, but that some people have such a faculty, I have had too much evidence to doubt.

*With a practical application of these discoveries, I have done.* All writers on masturbation, whom I have consulted, treat of it as being an acquired vice, but this I am confident is an error. It is a physiological vice, the result of a malorganization. When it is acquired, it never becomes inveterate, and marriage is the most appropriate remedy; but when it results from an abnormal organization, marriage greatly aggravates it. To such men a woman is not only as useless, but as objectional as would be a fifth wheel to a wagon. This condition results from a high endowment of the mesial or amatory third of the cerebellum, and a feeble endowment of the organs of sensation. Those having also a feeble endowment of the motory organs, the vice may induce inflammation and suppuration in the amatory organs, and then follows demensia and death. These may be rare results of the vice, but they do obtain. This truly unfortunate organization, more frequently than otherwise, results from physiologically incompatible marriage; and hence this vice is usually associated with a scrofulous diathesis, and hence phthisis is not an unfrequent sequel of this vice. It has been taught by some, that this vice is a cause of phthisis. An exciting cause it may be, I admit, but not the remote.

I have visited, probably, a majority of the lunatic asylums of our country, and found in many of them masturbators of both sexes. About 15 years ago, or a dozen years after making the preceding discoveries, I visited a lunatic asylum in one of the Southern States. The physician of the institution and myself had been fellow-students and graduates. He conducted me through the institution, and in our progress we entered a room in which were six male masturbators, with their hands bound behind them. I inquired of the physician the object of it? He answered, "To keep them from self-pollution." But do you expect by such treatment to restore them to society and usefulness? "No, Sir; but what else can be done? Their condition is not pathological, and hence they have no need of medicine." I rejoined, you are correct, Sir; their condition is physiological, and as physiology is a department of our profession, their condition is not beyond the resources of our profession. "What plan of treatment would you suggest?" Allow me to examine their heads, and I will respond. "Certainly, Sir; you can do so." I found all of them deficient in animal sensibility, and some of them in motor power. I explained to him my plan, thus:—procure a sufficient number of axes, wedges, and mauls; have a number of pretty large timber trees brought into the lot, minus the brush, and make these masturbators work them into convenient firewood; have them to begin their labor at daylight, and continue it till dark, and so closely too, as to make rest absolutely imperative; so that when they retire to their beds they will sleep instead of masturbating. By this labor their seminal glands will secrete less, and thus reduce their amatory irritation; the organs of sensation and motion will be more developed; and when by this labor the vicious habit is arrested, reduce their labor and provide them amusement, as music and dancing. By this plan, I am confident you may, in less than a year, restore them to their friends. This course will establish an equlibrium amongst the vital forces, which is a normal condition. This treatment will also remove the scrofulous diathesis from those of them who have it—and several of them have it.

He rejoined, "I fear your suggestion is impracticable with these youths—they are the sons of affluent planters, and they would seriously object to their sons being made to labor." I interrogated, Is not your will the law of the institution? He responded, "It is." I rejoined, as these youths were sent here for treatment, and as it is your duty to restore them if you can; then, Sir, as I conceive, it is your duty to teach those planters that they must conform to the laws of the institution, or take their sons out of it. He rejoined, "Have you ever made a trial of this treatment?" Not thoroughly, but to an extent, on negroes, that satisfied me that it will succeed. "Your treatment favorably impresses me; and as it is both my duty and desire to restore all who are sent here, I will adopt your suggestion, and inform you of the result." He continued, "I have several female masturbators here; what can I do with them?" I answered, make them sweat over the wash-tub, or over the floors, in scrubbing them or in brushing them. "Their mothers would never stand it." If I were in your place they would have to stand it, or take them home. In two months, he informed me by letter, that he had restored to their friends two of the masturbators whom I saw, and had a flattering prospect of restoring them all.

I have seen, I think, ten women under treatment for nymphomania; with nine of them the organs of sensation were large, but in the tenth, they were small; she, I suspect, was a masturbator and not a nymphomaniac. In the nine the amatory organs were not large. These observations have caused me to depart from authority in relation to the precise character of this affection. It is not, I think, a maniacal desire for amatory relief, but a maniacal desire for the pleasure that sexual commerce affords them.

The faculties of sensation, motion, and amativeness I denominate the vital forces or faculties; and the abnormal development of their organs in relation to each other accounts for all the vices that obtain in the relation the respective sexes hold to each other, and very much of the abnormal condition of these organs results from the physiological incompatibility of progenitors. I saw in the New Orleans Hospital, a eunuch, the result of incompatible marriage. The individual had a penis, but neither a beard nor testicles. The intellectual organs, those of the human sentiments and of sensation and motion, were respectably developed, but the mesial third of the cerebellum—the organs of the "instinct of reproduction" did not constitute a portion of the cerebellum. This individual was exclusively a neuter—had no instinct of sex, and could form no conception of it. I have the skull of this individual—it is a very remarkable illustration of human monstrosity.

A feeble development of the motory centre is, in my opinion, that condition which constitutes the scrofulous diathesis; and as I know the motory centre can be rapidly developed by exercise, consequently, I infer that judicious exercise in the open air, began in infancy, would save 75 per centum of those who otherwise would prematurely die of some scrofulous form of disease. The motory centre is, I doubt not, the source of involuntary respiration, and hence it is that a high endowment of this centre cannot coëxist with the scrofulous diathesis. Physiologists, I believe, regard the medulla oblongata as being the respiratory centre. But this conclusion requires some qualification; a high endowment of this centre is an indicator of the scrofulous diathesis. It is at the most, therefore, the source, only, of voluntary respiration.

